# Postoperative Sequelae and Complications of Alveolar Ridge Augmentation With Simultaneous Implant Placement. A Retrospective Cohort‐Based Predictive Risk Modelling

**DOI:** 10.1111/jcpe.70196

**Published:** 2026-09-13

**Authors:** Muhammad H. A. Saleh, Hend Abulatifa, Francesco Fanelli, Abdusalam E. Alrmali, Asmaa W. Issa, Hom‐Lay Wang, Giuseppe Troiano

**Affiliations:** ^1^ Department of Periodontics and Oral Medicine, School of Dentistry University of Michigan Ann Arbor Michigan USA; ^2^ Department of Clinical and Experimental Medicine University of Foggia Foggia Italy; ^3^ Department of Oral Medicine, Oral Pathology, Oral and Maxillofacial Surgery University of Tripoli School of Dentistry Tripoli Libya

**Keywords:** Alveolar ridge augmentation, dental implants, postoperative complications, risk factors

## Abstract

**Aims:**

To quantify the incidence and develop predictive models for postoperative sequelae and complications (POSC) and the need for additional grafting after alveolar ridge augmentation with simultaneous implant placement (SARA).

**Methods:**

This longitudinal retrospective cohort included adults with ridge deficiencies who underwent SARA with implant placement from 2019 to 2024 at the University of Michigan Graduate Periodontics Clinic. One site per patient was analysed. Complications were classified according to the severity of consequences and need for further intervention. Univariable and multivariable logistic regression models were performed with stepwise refinement. Firth penalized logistic regression was used when sparse data/separation were present. Model discrimination (area under the curve, AUC) and calibration (Hosmer–Lemeshow, HL) were assessed; ROC‐derived thresholds were used to report operating characteristics.

**Results:**

Three‐hundred and ninety‐one patients were included. Systemic comorbidities were documented in 48.6%. Postoperative sequelae occurred in 31.5% of patients, and complications occurred in 25.8%. Membrane exposure was associated with vertical/combined defects (adjusted odds ratio [aOR] 2.52; 95% CI: 1.50–4.23). Microsurgical technique was associated with lower odds of incision‐line dehiscence (aOR 0.76; 95% CI: 0.57–1.00; *p* = 0.049). Prolonged swelling was associated with flap extension (aOR 1.21 per tooth site; 95% CI: 1.04–1.39). Additional grafting occurred in 6.14% and was associated with clinical attachment loss (CAL ≥ 5 mm on adjacent tooth [OR 14.07; 3.79–52.18; *p* < 0.001]), current smoking (OR 5.11; 1.38–18.95; *p* = 0.015) and long‐term systemic corticosteroid use (OR 1.61; 1.04–2.51; *p* = 0.035) (AUC 0.744; HL *p* = 0.5103).

**Conclusion:**

Postoperative sequelae were more frequent than true complications, and the need for additional grafting was uncommon. Complications were mainly associated with defect morphology and surgical technique rather than systemic comorbidities.

## 1 Introduction

Alveolar ridge augmentation (ARA) is performed either as a two‐stage (staged) procedure or simultaneously with implant placement (Yu et al. 2023). The simultaneous strategy has gained growing clinical interest, as it allows ARA and implant placement in a single surgical session, thereby reducing treatment time and the number of surgical interventions (Kohavi 2000; Zhang et al. 2019).

Systematic reviews have shown that SARA with implant placement can achieve high implant survival rates (80–100%) and substantial defect reduction (Donos et al. 2008; Jiménez Garcia et al. 2017; Merli et al. 2016; Sanz‐Sánchez et al. 2015). ARA performed at the time of implant placement may involve different regenerative approaches depending on the defect morphology and surgical requirements. These include guided bone regeneration (GBR) with resorbable or non‐resorbable membranes, titanium‐reinforced membranes or meshes, particulate grafting techniques and block graft procedures. Despite the widespread use of these approaches, evidence on predictors of postoperative complications remains limited.

*Adverse events* are any unfavourable and unintended signs, symptoms, diseases or abnormal findings occurring after a treatment or procedure, such as a *negative outcome* related to the procedure (e.g., postoperative infection) or one that is not related to it (e.g., postoperative rash from antibiotics prescribed after surgery) (Technical Requirements For Pharmaceuticals 2025). Clavien and Dindo further divided negative outcomes into *complications*, which are any deviation from the standard postoperative healing path, *postoperative sequelae*, which are predictable after‐effects of the surgical procedure and *failure to cure*, which is when a properly executed procedure fails to meet its primary objective (Dindo et al. 2004). Complications are well documented after ARA and may arise from multifactorial interactions between patient‐ and site‐related factors (Shen et al. 2022). Patient‐related factors such as smoking and uncontrolled diabetes impair angiogenesis and fibroblast activity, may delay healing and increase dehiscence and infection risk (Tawil et al. 2008). Site‐related factors, including defect size and morphology, soft‐tissue quality, and flap tension, are likewise strongly associated with higher complication rates (Donos et al. 2023; Schliephake 2022). The other components of negative outcomes, such as postoperative sequelae and failure to cure, are, on the other hand, rarely reported (Gallo and Diaz‐Baez 2019).

Although GBR generally shows fewer complications than other ARA approaches (Urban et al. 2019), the simultaneous strategy remains technique‐sensitive. Because implant placement and bone augmentation are performed within a single surgical procedure, achieving primary wound stability becomes particularly critical (Saleh, Calatrava, et al. 2026; Simion et al. 1994). Membrane exposure rates of approximately 22.7% have been reported for GBR performed simultaneously with implant placement (Thoma et al. 2019).

Several studies have evaluated the outcomes and complication rates of SARA, but most have focused solely on implant survival and success (Bazrafshan and Darby 2014; Rammelsberg et al. 2012; Shen et al. 2022; Tatli et al. 2025). Also, a few studies have evaluated predictors of complications specifically in SARA procedures across different augmentation techniques (Kalani et al. 2026; Milinkovic and Cordaro 2014). Therefore, the primary outcome of this study was to quantify the incidence of postoperative sequelae and complications (POSC) following SARA. The secondary outcome was to assess the need for additional bone and tissue augmentation, and to develop predictive models evaluating the contribution of local and systemic factors to (a) complication risk and (b) the need for additional grafting.

## 2 Materials and Methods

### 2.1 Study Design and Population

This retrospective cohort study included all adult patients (> 18 years) with ridge deficiencies who underwent SARA between 2019 and 2024 at the Graduate Periodontology Clinic, University of Michigan School of Dentistry (Ann Arbor, MI, USA). Cases were systematically screened and extracted by two calibrated examiners (H.A. and A.A.). Disagreements were resolved through discussion, with a senior author (M.H.A.S.) adjudicating when consensus could not be reached.

### 2.2 Ethical Considerations and Consent (Appendix)

### 2.3 Eligibility Criteria (Appendix)

### 2.4 Data Collection and Variables (Appendix)

### 2.5 Study Outcomes

#### 2.5.1 Incidence of Postsurgical Complications (Primary Outcome)

The primary outcome of this study was the incidence of POSC after SARA. Building on previous key publications (Fontana et al. 2011; Gallo and Diaz‐Baez 2019; Li and Wang 2008; Sanz‐Sanchez et al. 2022; Vroom et al. 2022), postoperative complications were further organised using a study‐specific classification that grouped events by severity of consequences and need for further intervention. The acronym ‘WOUND’ comprised five domains:

**W**: Early wound disturbances: Included early inflammatory or soft‐tissue healing disturbances, such as erythema, tenderness, swelling and ulceration, persistent at 2 weeks.

**O**: Wound **O**pening: referred to incision line dehiscence with or without the underlying membrane or device exposure.

**U**: sUppuration/p**U**rulence: included early and late postoperative infection.

**N**: **N**on‐integration: included early implant failure and graft failure.

**D**: **D**amage to adjacent structures: damage to adjacent structures, specifically nerve injury or hypermobility to adjacent teeth with loss of attachment.

In the present study, postoperative sequelae consisted of the incidences reported under ‘W’, and complications consisted of the incidences reported under ‘O’, ‘U’, ‘N’ or ‘D’.

#### 2.5.2 Need for additional bone grafting (Secondary outcome) (Appendix)

#### 2.5.3 Need for additional tissue grafting (Secondary outcome) (Appendix)

#### 2.5.4 Prediction models (Secondary outcome) (Appendix)

### 2.6 Handling of missing data (Appendix)

### 2.7 Statistical Analysis

All analyses were performed in Stata/SE 16.1 (StataCorp, College Station, TX, USA). Baseline characteristics of the analysed cohort were summarised as mean ± SD for continuous variables and as frequencies for categorical variables. Univariable logistic regression models were fitted for each candidate predictor and outcome, and odds ratios (ORs) with 95% confidence intervals (CIs) and *p‐*values were reported. Reference categories were absence/non‐exposure for binary variables and the first clinically coded category for categorical variables unless otherwise specified; continuous, count or ordered variables were modelled per unit increase in the coded variable. All available demographic, behavioural, systemic, site‐related and procedure‐related variables were initially screened as candidate variables or potential confounders. Variables showing a statistically meaningful or clinically relevant association with the outcome, together with biologically plausible confounders, were subsequently considered for multivariable modelling. Analyses were conducted at the surgical‐site level, and postoperative complication categories were evaluated separately according to their predefined diagnostic criteria. Where complete or quasi‐complete separation or very sparse cells were detected, estimates were obtained using penalised (Firth) logistic regression to ensure finite parameter estimates.

Given the limited number of events for several outcomes, parsimonious reduced models were preferred over fully saturated multivariable models to avoid over‐parameterisation and improve model stability and interpretability. Bootstrap resampling (1000 replications) was used to assess the stability of model discrimination within the development cohort. Formal interaction terms were not included because the analyses were intended to remain exploratory and parsimonious given the limited number of events for several outcomes. Model refinement was applied when appropriate using stepwise selection to limit model complexity and enhance clinical interpretability, retaining only predictors with an independent contribution (retention *p* < 0.05) and removing redundant or non‐estimable terms. Variables were excluded from the final models when they showed no independent contribution, produced unstable estimates, were affected by sparse data or complete/quasi‐complete separation, showed collinearity or increased the risk of overfitting.

To further assess the robustness of the main multivariable findings, additional sensitivity‐adjusted models were performed using the predictors retained in the final multivariable models and further adjusting for key clinically relevant covariates, including age, sex, never‐smoked status, diabetes and systemic steroid use, where not already included in the model. At the end of the process, two predictive models were developed: for each model, discrimination was quantified by the area under the ROC curve (AUC) with 95% CIs, and calibration was evaluated using the Hosmer–Lemeshow (HL) goodness‐of‐fit test. An optimal classification threshold was selected from the ROC output to balance sensitivity and specificity, and sensitivity, specificity, accuracy, LR+, and LR− at that threshold were reported. A *p* < 0.05 was considered statistically significant.

## 3 Results

### 3.1 Descriptive Characteristics of the Study Population

The analysed cohort comprised 391 patients (391 sites) with a median age of 62 (IQR 50–68) years and a female proportion of 53.9%. The selection process is depicted in Figure S1. At the time of surgery, 44 patients (11.25%) were current smokers and 190 patients (48.6%) had one or more systemic comorbidities, most notably, rheumatoid arthritis (87, 22.3%), hypertension (77, 19.69%), diabetes mellitus (59, 15.1%) and history of cancer (35, 8.95%) (Figure 1A,B). Among the grafted sites, the posterior mandible accounted for the largest share (177, 45.3%), followed by the posterior maxilla (95, 24.3%), anterior maxilla (91, 23.3%) and anterior mandible (28, 7.2%) (Tables S1 and S2). The complete demographic and clinical profile of the study population is presented in Table 1.

**FIGURE 1:**
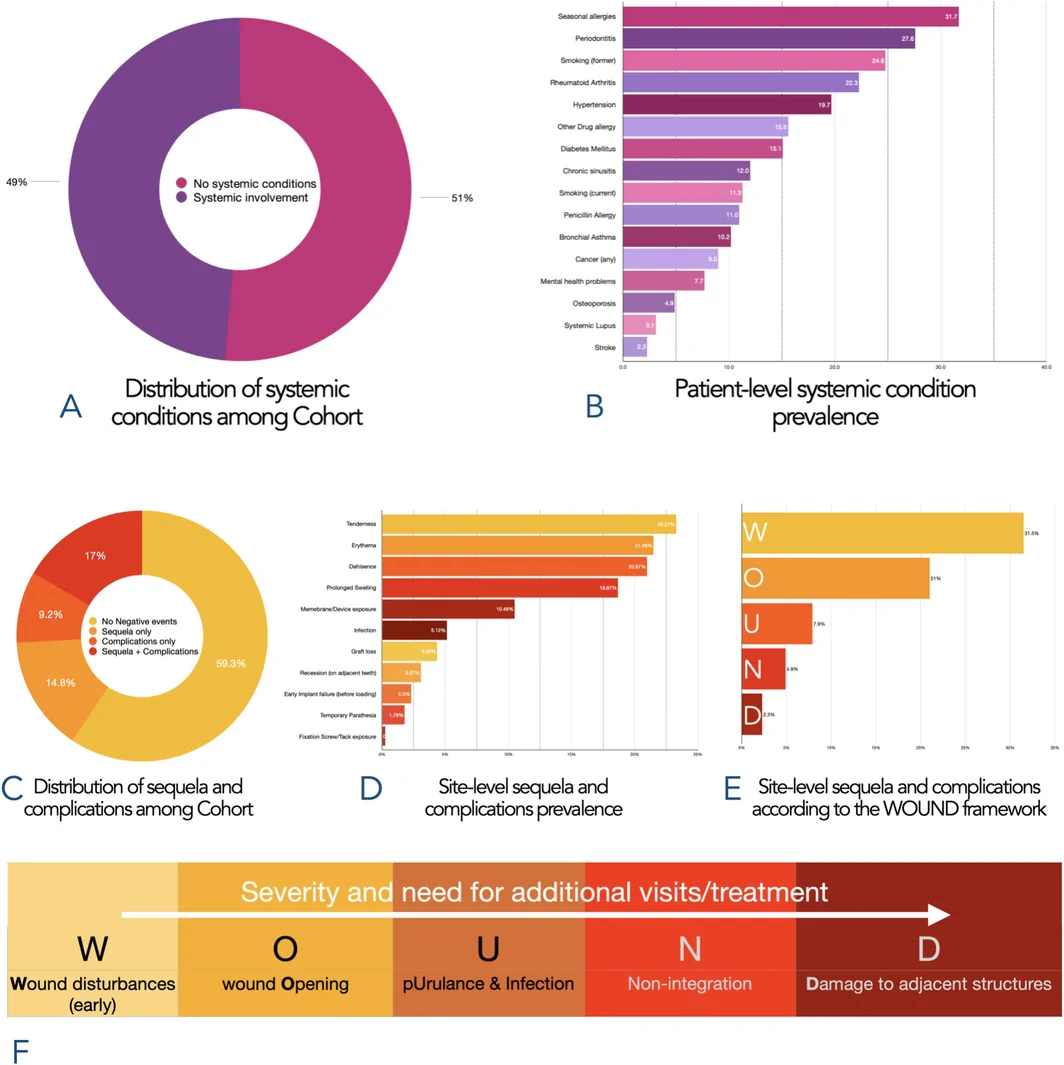
Cohort distributions of systemic conditions and postoperative events. (A) Distribution of patients with and without systemic comorbidities. (B) Patient‐level prevalence of specific systemic conditions. (C) Overall distribution of patients with versus without POSC. The total frequency does not add up to 100% since the same patient/site can have more than one sequela/complication (D). Site‐level prevalence of individual POSC. (E) Site‐level distribution of POSC according to the WOUND framework. (F) The WOUND framework, a severity‐based classification of POSC based on increasing clinical severity and need for additional visits/treatment: W, wound disturbances (early; erythema, tenderness, swelling); O, wound/Incision line opening; U, suppuration and infection; N, non‐integration (early implant failure, graft failure/bone sequestration); and D, damage to adjacent structures (nerve damage, damage to adjacent teeth with attachment loss).

**TABLE 1:** Demographic and clinical characteristics of patients in the SARA group.

	Variable	*N* (%)
	Total number of patients	391
	Total number of Sites	391
	Age median (IQR)	62 (50–68)
	Male/female ratio	183: 208 (46.1%:53.9%)
Systemic conditions	≥ 1 comorbidity	190 (48.6%)
	Diabetes	59 (15.1%)
	Periodontitis	108 (27.6%)
	Smoking (current)	44 (11.2%)
	< 10 cigarettes/day:	27(6.9%)
	≥ 10 cigarettes/day:	17(4.3%)
	Smoking (former)	97 (24.8%)
	Arthritis	87 (22.3%)
	Asthma	40 (10.2%)
	Dialysis	0 (0.0%)
	Stroke	9 (2.3%)
	Sinus problems	47 (12.0%)
	Osteoporosis	19 (4.9%)
	History of cancer (any)	35 (8.95%)
	Mental health problems	30 (7.7%)
	Hypertension	77 (19.7%)
	Systemic lupus erythematosus	12 (3.1%)
Allergies	Penicillin	43 (11.0%)
	Erythromycin	9 (2.3%)
	Codeine	20 (5.1%)
	Latex	9 (2.3%)
	Seasonal	124 (31.7%)
	Local anaesthetics	1 (0.3%)
	Food (misc.)	22 (5.6%)
Type of defect	Combined	20 (5.1%)
	Horizontal	296 (75.7%)
	Vertical	75 (19.2%)
Anatomic location of ARA	Anterior mandible	28 (7.2%)
	Anterior maxilla	91 (23.3%)
	Posterior mandible	177 (45.3%)
	Posterior maxilla	95 (24.3%)
Medications taken by patients at the time of surgery	Chemotherapy	11 (2.8%)
	Systemic steroids	4 (1%)
	Antihypertensives	76 (19.4%)
	Statins	51 (13.0%)
	Blood thinners	31 (7.9%)
	Antidiabetics	20 (5.1%)
	Antidepressants	29 (7.4%)
	Anticonvulsant	14 (3.6%)
Perioperative therapeutics	Sedation at the time of procedure	61 (15.6%)
	Antibiotic therapy after the procedure	389 (99.5%)

### 3.2 Incidence of POSC

Postoperative sequelae ‘W’ were encountered in 123 (31.5%) patients, and complications ‘O’, ‘U’, ‘N’ and ‘D’ were found in 101 (25.8%) patients. The total number of patients who encountered any postoperative sequelae or complications was 159 (40.66%) (Figure 1C,D).

When divided according to the WOUND framework (Figure 1E,F), postoperative events were distributed predominantly towards the lower severity end of the spectrum. W was the most common, occurring in 31.5% of sites, followed by incision‐line opening (O) in 21.0%, purulence/infections (U) in 7.9% of sites, graft and implant non‐integration (N) in 4.9%, and damage to adjacent structures (D) in 2.3% (Figure 1E,F). Results of the univariable analyses and the adjusted ORs (aORs) from the multivariable models are presented in Table 2. In general, complications and the need for additional grafting were significantly associated to clinical and site‐specific variables. Descriptive distributions of postoperative sequelae, complications and secondary interventions across systemic, medication‐related and site‐level variables are shown in Figures 2, 3, 4 and Appendix 3.2 in the Supporting Information.

**TABLE 2:** Univariate and multivariate logistic regression analysis of variables for POSC, and the need for additional grafting after SARA.

		Univariate logistic regression			Multivariate logistic regression		
Variables		OR	95% CI	*p*	OR	95% CI	*p*
Postoperative sequela and complications							
Membrane exposure	Ti‐reinforced membrane/Ti mesh (*n* = 3)	18.79	1.66–212.61	0.018	—	—	—
	Vertical/combined defects (*n* = 95)	2.31	1.42–3.78	0.001	2.52	1.50–4.23	< 0.001
	ARA location, overall effect	—	—	0.0218	—	—	**0.024**
Incision‐line dehiscence	Vertical/combined defects (*n* = 95)	1.70	1.14–2.54	0.010	1.54	1.01–2.33	0.044
	Microsurgical techniques (*n* = 17)	0.74	0.56–0.97	0.029	0.76	0.57–1.00	**0.049**
Prolonged swelling	Sedation (*n* = 61) (yes vs. no)	2.37	1.28–4.41	0.006	2.31	1.09–4.87	0.028
	Smoking ≥ 10 cigarettes (*n* = 17)	1.52	1–2.31	0.048	—	—	—
	Flap extension (1–14 teeth)	1.21	1.04–1.40	0.010	1.21	1.04–1.39	**0.012**
	Sex (Male/female = 183: 208)	0.44	0.25–0.76	0.003	—	—	—
Need for additional bone grafting							
	Ti‐reinforced membrane/Ti mesh (*n* = 3)	31.99	2.79–366.69	0.005	—	—	—
	CAL on adjacent teeth (*n* = 12)	13.00	3.77–44.81	< 0.001	14.07	3.79–52.18	< 0.001
	Smoking (current) (*n* = 44)	4.13	1.21–14.13	0.024	5.11	1.38–18.94	0.015
	Long‐term corticosteroid use (*n* = 4)	1.56	1.03–2.37	0.037	1.61	1.04–2.51	0.035

*Note:* Reference categories are absence/non‐exposure for binary predictors and the first clinically coded category for categorical variables, unless otherwise specified. ORs for continuous and count variables refer to a unit increase. For ARA location, the global *p*‐value was obtained using a likelihood‐ratio test in the univariable analysis and a joint Wald test in the multivariable analysis. Only Significant variables were retained.

**FIGURE 2:**
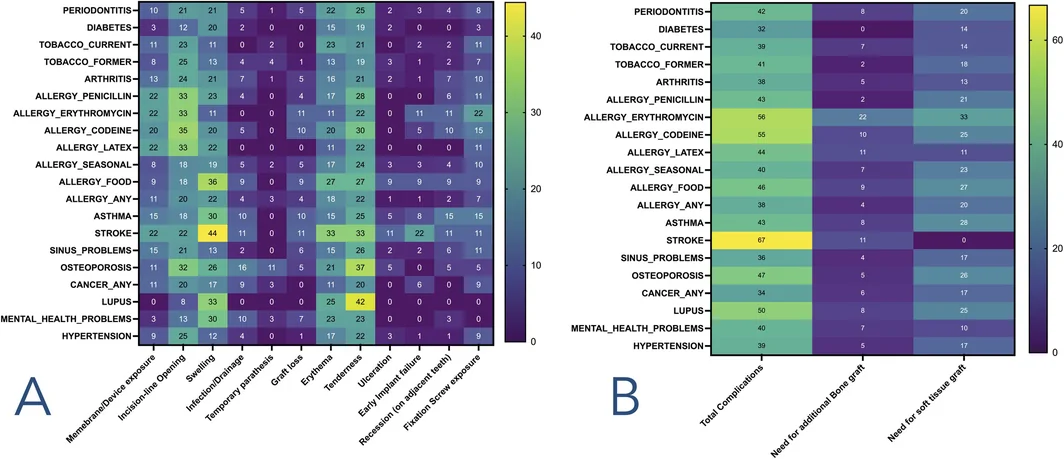
Heatmap of associations between systemic conditions and POSC. (A) Patient‐level distribution of complications across systemic comorbidities. (B) Patient‐level distribution of overall complications and secondary interventions (need for additional bone grafting and need for soft‐tissue grafting) across systemic comorbidities; higher color intensity indicates higher prevalence.

**FIGURE 3:**
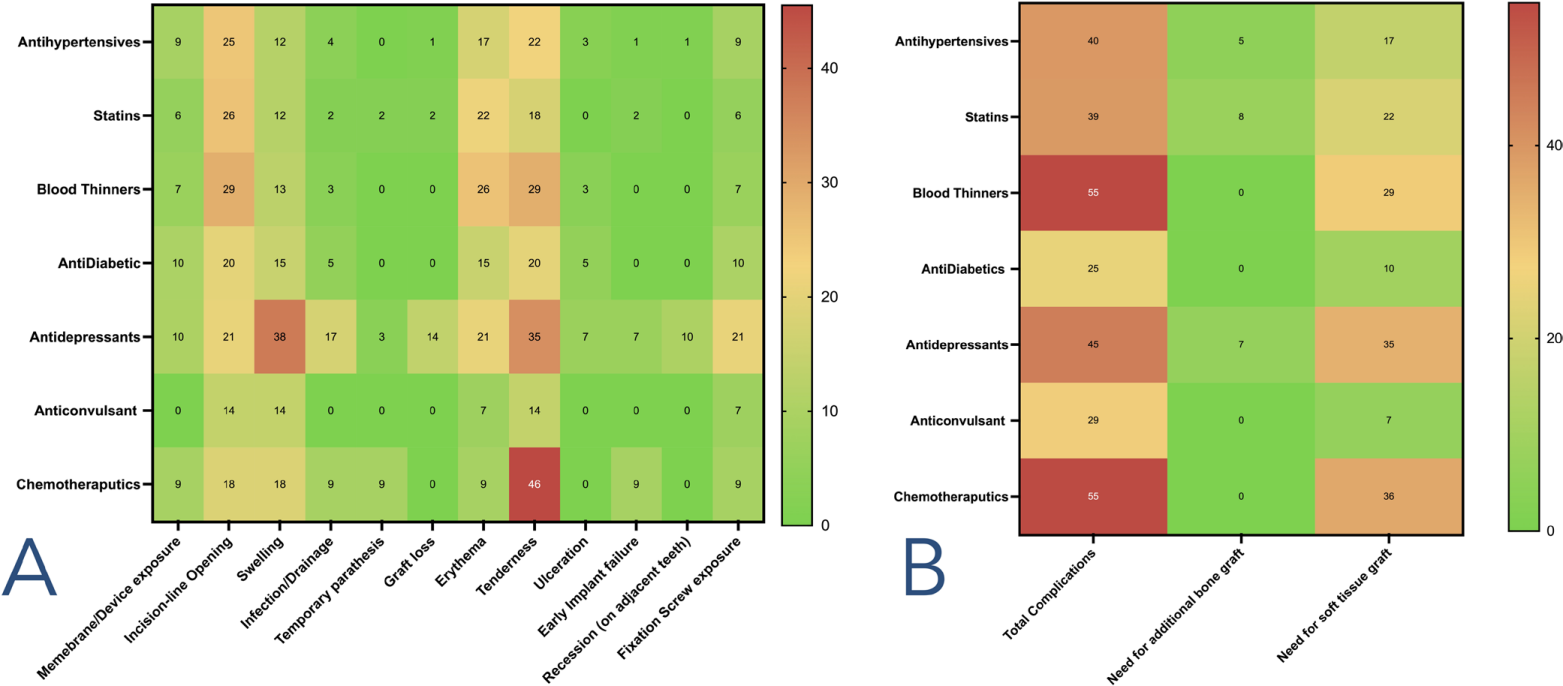
Heatmap of associations between medication classes and POSC. (A) Patient‐level distribution of complications across medication categories. (B) Patient‐level distribution of overall complications and secondary interventions (need for additional bone grafting and need for soft‐tissue grafting) across medication categories; higher colour intensity indicates higher prevalence.

**FIGURE 4:**
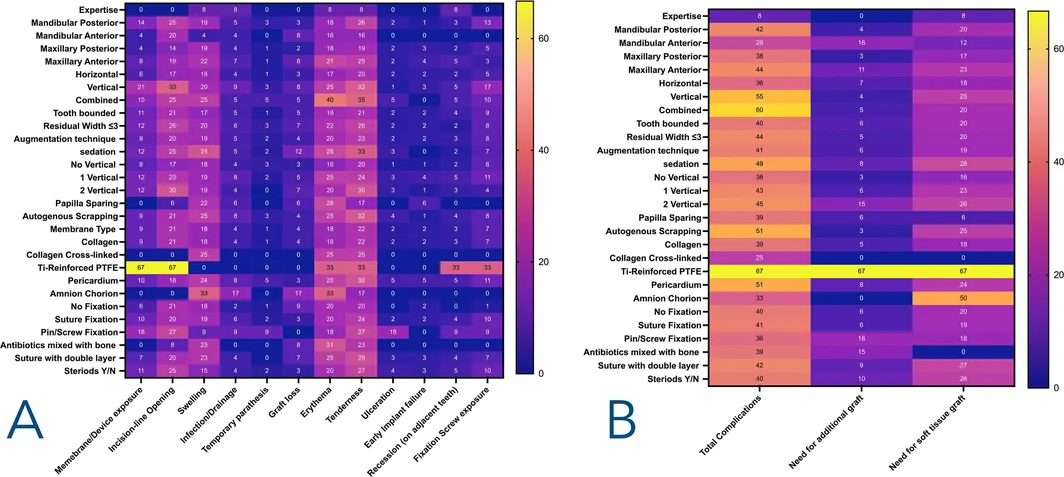
Heatmap of associations between clinical/site‐level factors and POSC. (A) Patient‐level distribution of POSC across operator, anatomic, defect‐, flap‐, material‐ and fixation‐related variables. (B) Patient‐level distribution of overall complications and secondary interventions (need for additional bone grafting and need for soft‐tissue grafting) across the same clinical/site‐level variables; higher colour intensity indicates higher prevalence.

#### 3.2.1 Membrane Exposure

Membrane exposure was primarily associated with the location and configuration of the defect reconstructed. Univariable analyses showed that the use of titanium meshes/titanium‐reinforced PTFE membranes was associated with approximately 19‐fold higher odds of exposure (OR 18.79; CI: 1.66–212.61; *p* = 0.018). Smaller yet still significant increases were also observed for vertical or combined defects (OR 2.31; CI: 1.42–3.78; *p* = 0.001). When ARA location was modelled as a nominal four‐category variable, it showed a significant overall association (*p* = 0.0218), but none of the individual regions differed significantly. In the multivariable model, vertical/combined defects remained associated with exposure (aOR 2.52; 95% CI: 1.50–4.23; *p* < 0.001), and ARA location remained significant as an overall factor (*p* = 0.024) with no individual comparisons reaching significance from the maxillary anterior reference: maxillary posterior aOR 0.55 (95% CI: 0.15–1.98; *p* = 0.359), mandibular anterior aOR 0.61 (95% CI: 0.07–5.38; *p* = 0.656) and mandibular posterior aOR 2.37 (95% CI: 0.95–5.91; *p* = 0.063). The overall model was significant (likelihood ratio *χ* ^2^(4) = 20.91; *p* = 0.0003; *n* = 365) (Table 2).

#### 3.2.2 Dehiscence

Incision‐line dehiscence was chiefly influenced by defect morphology and flap extensiveness vertical/combined defects exhibited a 70% greater likelihood of dehiscence (OR 1.70; CI: 1.14–2.54; *p* = 0.010), whereas adopting a microsurgical technique lowered the odds by roughly one‐quarter (OR 0.74; CI: 0.56–0.97; *p* = 0.029).

Both variables retained independent weight in the multivariable model. Vertical/combined ARA increased the odds by 54% (aOR 1.54; 95% CI: 1.01–2.33; *p* = 0.044), whereas microsurgical techniques were associated with lower odds of dehiscence, reducing the risk by roughly one‐quarter (aOR 0.76; 95% CI: 0.57–1.00; *p* = 0.049) (Table 2).

#### 3.2.3 Infection (Appendix)

#### 3.2.4 Prolonged swelling (Appendix)

#### 3.2.5 Sensitivity‐Adjusted Analyses

Additional sensitivity‐adjusted multivariable models were studied using the predictors retained in the final multivariable analyses, with further adjustment for age, sex, never‐smoked status, diabetes and systemic steroid use, where not already included in the model. The direction and magnitude of the main associations remained largely unchanged. Detailed results are reported in Tables S4 and S5.

### 3.3 Need for Additional Bone Grafting

Only 24 patients (6.14%) of 391 required additional bone grafting at implant uncovering. Univariable logistic analysis showed that titanium meshes were strongly associated with the need for a second graft compared with resorbable membranes (OR 31.99; CI: 2.79–366.69; *p* = 0.005). CAL ≥ 5 mm on adjacent teeth was another critical factor (OR 13.00; CI: 3.77–44.81; *p* < 0.001). Smoking and systemic steroid therapy also increased the probability of an additional grafting (Table 2).

When these variables were entered simultaneously into the multivariable model, CAL ≥ 5 mm on adjacent teeth showed the strongest association (OR 14.07; CI: 3.79–52.18; *p* < 0.001). Smoking history and systemic steroid use retained a meaningful effect (OR 5.11; CI: 1.38–18.94; *p* = 0.015) and (OR 1.61; CI: 1.04–2.51; *p* = 0.035), respectively (Table 2).

### 3.4 Need for Additional Soft Tissue Grafting (see Appendix A in the Supporting Information)

Two multivariable logistic regression models were developed to estimate the risk of two clinically relevant events: membrane exposure and the subsequent need for additional bone graft.

### 3.5 Predictive Models

#### 3.5.1 Model A (Complications)

For membrane exposure, the model included two statistically significant variables. Defect morphology (vertical/combined defects) was associated with a higher risk of membrane exposure (aOR 2.52; 95% CI 1.50–4.23; *p* < 0.001). ARA location showed a significant overall association with exposure (global Wald *χ* ^2^(3) = 9.44; *p* = 0.024). Compared with the maxillary anterior region, the maxillary posterior region showed 0.55 for the adjusted OR (95% CI: 0.15–1.98; *p* = 0.359), the mandibular anterior region 0.61 (95% CI: 0.07–5.38; *p* = 0.656) and the mandibular posterior region 2.37 (95% CI: 0.95–5.91; *p* = 0.063). The model showed satisfactory calibration (HL *χ* ^2^ = 4.20; *p* = 0.379he 8) and moderate discrimination, with an AUC of 0.708 (95% CI: 0.627–0.788). The optimal probability threshold was 0.0590, corresponding to a sensitivity of 83.78%, specificity of 45.12%, overall accuracy of 49.04%, LR+ of 1.53 and LR− of 0.36 (Figure 5A).

**FIGURE 5:**
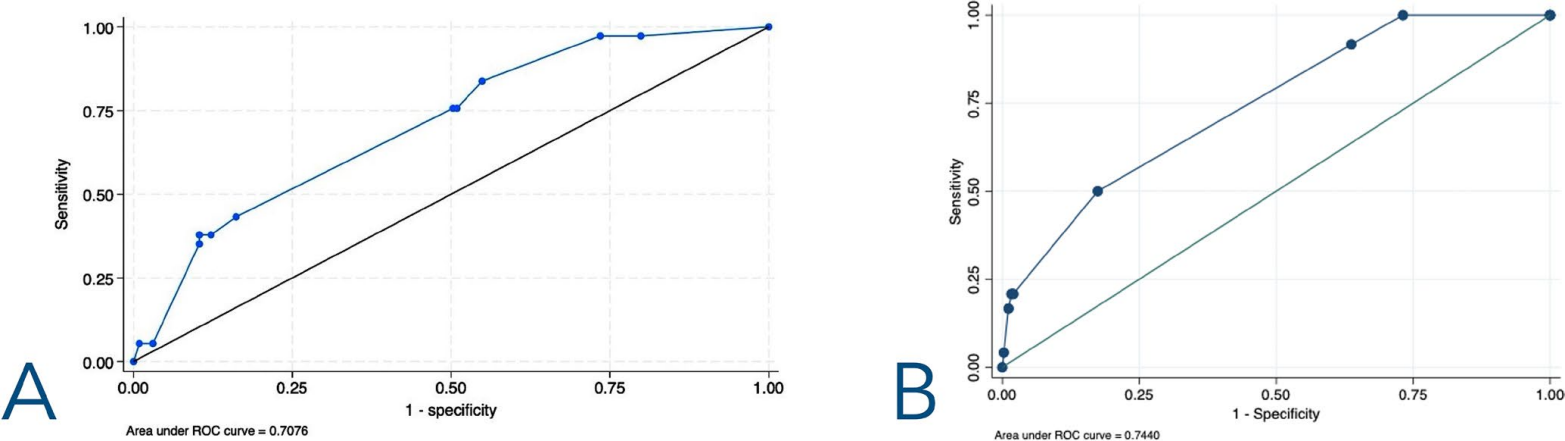
Predictive models for complications (A) and need for additional grafting (B) following SARA. (A) Receiver operating characteristic curve for the revised multivariable model predicting membrane/device exposure. The model included defect type and ARA location modelled as a nominal four‐category variable. The model had an AUC of 0.708 (95% CI: 0.627–0.788), HL *χ* ^2^ = 4.20 (*p* = 0.3798), sensitivity of 83.78% and specificity of 45.12% at the optimal threshold of 0.0590. (B) ROC curve of the model predicting the need for additional bone grafting, with CAL ≥ 5 mm on adjacent teeth, smoking habit and corticosteroid use as independent predictors. This model showed better discriminative ability (AUC 0.744; 95% CI: 0.655–0.833), HL *χ* ^2^ = 2.31 (*p* = 0.5103), sensitivity of 50.0% and specificity of 82.6% at the optimal threshold.

#### 3.5.2 Model B (Need for Additional Grafting)

The second model, developed to predict the need for an additional bone graft, included three variables: presence of advanced CAL ≥ 5 mm on adjacent teeth, smoking and corticosteroid use. All three were statistically significant predictors. CAL ≥ 5 mm on adjacent teeth was strongly associated (OR 14.07; 95% CI: 3.79–52.18; *p* < 0.001), smoking had an odds ratio of 5.11 (95% CI: 1.38–18.95; *p* = 0.015) and corticosteroid use had an odds ratio of 1.61 (95% CI: 1.04–2.51; *p* = 0.035). This model showed good calibration (HL *χ* ^2^ = 2.31, *p* = 0.5103) and slightly higher discriminative ability than Model A, with an AUC of 0.744 (95% CI: 0.655–0.833). As shown in Figure 5B, the optimal classification threshold was 0.1280, corresponding to a sensitivity of 50.00% and a specificity of 82.57%, with an overall accuracy of 80.30%, an LR+ of 2.87 and an LR− of 0.61 (Table S3).

## 4 Discussion

### 4.1 Main Findings

In this cohort of 391 patients undergoing SARA, membrane exposure and incision‐line dehiscence were the most frequent negative outcomes and were primarily associated with defect location, morphology and the augmentation approach. Additional bone grafting was associated with CAL > 5 mm on adjacent teeth, smoking and systemic corticosteroid therapy. The predictive models for membrane exposure and secondary grafting demonstrated moderate discriminative performance (AUCs of 0.708 and 0.744); however, their predictive accuracy remains limited, and further refinement and external validation are required before clinical implementation can be considered.

Previous classic articles have identified smoking, wearing removable prostheses and defect location as independent variables associated with surgical complications, including incision‐line dehiscence (Blanco et al. 2005; Zitzmann et al. 1999). This cohort confirmed associations between smoking and an increased risk of prolonged swelling and a significantly higher need for additional bone grafting (aOR 5.11); however, no statistically significant association was found with the overall complication rate. This can be attributed to two reasons. First, the ‘combined postoperative event rate’ included both postoperative complications as well as sequelae. Sequelae naturally occurred more frequently across the entire cohort regardless of smoking status. This may have diluted the statistical power to detect smoking as a primary driver of the overall rate. Second, current smokers represented only 11.25% of the cohort (44/391), limiting the statistical power to detect a significant association with the composite complication outcome.

In hindsight, events such as swelling and erythema inflate the complication rate if counted as a ‘complication’ and are better considered ‘postoperative sequelae’ if deemed to require no clinician intervention (Dindo et al. 2004). However, the overall postoperative sequelae and complication rate of 40.66% observed in this study must be interpreted in the context of an academic graduate training clinic; therefore, the event rates may not generalise directly to other clinical settings. All surgical procedures were performed by residents under faculty supervision.

Although postoperative swelling after ARA is typically self‐limiting, it may impair patient comfort and delay functional recovery by compromising vascularity and creating a microenvironment more susceptible to bacterial colonisation (Leblebicioglu and Tatakis 2023). In this cohort, each additional tooth included within the surgical flap increased the risk of postoperative swelling by 21%, while sedation was associated with 131% higher odds. However, sedation may have acted as a marker of surgical complexity, longer surgical duration, greater flap manipulation or more extensive procedures rather than as a direct biological cause of swelling.

The need for additional grafting is not routinely reported as an outcome in ARA studies (Cosyn et al. 2024; Fiorellini et al. 2026), although it has recently been recognised as a key domain in the Bone Augmentation Core Outcome Set and Measurement (BA‐COSM) (Tonetti et al. 2023). Higher rates of implant failure were also observed in defects requiring additional grafting (Rammelsberg et al. 2012). Repeat grafting in this study was strongly associated with CAL ≥ 5 mm on adjacent teeth, systemic corticosteroid therapy and smoking history. Although ARA outcomes have not been specifically reported in individuals with long‐term corticosteroid therapy, early implant failure rates may serve as a practical proxy for impaired bone‐healing capacity and may help estimate the risk of compromised regeneration (Hyldahl et al. 2025). That said, because additional bone grafting occurred in only 6.14% of patients, the corresponding multivariable model should be interpreted with caution, as the limited number of events may have reduced the statistical power and stability of the estimated associations. Finally, because long‐term corticosteroid use was documented in very few patients, this association with the need for additional grafting should be merely considered hypothesis‐generating.

### 4.2 Limitations

Reliance on self‐reporting for variables such as smoking introduces the potential for misclassification. SARA was performed with different biomaterials, membranes and devices, each of which may have a distinct complication profile. Accordingly, the presented models should be interpreted as reflecting the average risk of the overall study population rather than technique‐specific (Kalani et al. 2025). This is particularly relevant for titanium‐reinforced devices, which showed a strong univariable association with membrane exposure but were represented by very few cases and did not remain in the adjusted model (Urban et al. 2025). Under these conditions, non‐retention in multivariable analysis likely reflects sparse‐data limitations and limited estimability rather than evidence of no effect. Although candidate predictors were selected based on clinical relevance and biological plausibility, the use of univariable screening followed by stepwise multivariable refinement may still have increased the risk of model instability, false‐positive findings and overestimation of some associations. Furthermore, bootstrap validation was performed on the final reduced models and, therefore, may not have fully accounted for the optimism introduced during this variable selection process.

No patient‐reported outcome measures were collected, which undermines our proposed classification system (Braun et al. 2025). The decision to perform additional grafting (bone or soft tissue) was inherently based on clinical judgment and therefore involved a high degree of subjectivity (Saleh, Sabri, et al. 2026; Saleh, Schwarz, et al. 2026). Our reported protective effect of microsurgical technique against incision‐line dehiscence should be interpreted with caution. In this graduate training setting, adoption of a microsurgical approach is almost certainly not randomly distributed and may be clustered with other favourable operator‐related characteristics. As such, the microsurgical technique may have functioned partly as a proxy for clinical finesse or experience. In addition, because multiple procedures were performed by the same resident operators within a graduate training environment, observations may not have been fully independent. The absence of clustering adjustment (e.g., mixed‐effects modelling or cluster‐robust standard errors) may therefore have resulted in underestimated standard errors and optimistic confidence intervals.

Finally, in the present study, specific defect criteria were not identified. A dehiscence versus fenestration, as well as the dimensions of dehiscence defects, may substantially influence the likelihood of requiring additional grafting.

## 5 Conclusion

Within the limitations of this cohort study, postoperative sequelae were more frequent than complications of SARA. Wound‐opening complications were mainly associated with defect morphology, while prolonged swelling was associated with flap extension and sedation. Additional bone grafting was uncommon and was associated with adjacent‐tooth CAL, smoking and long‐term corticosteroid use. The internally developed models require external validation before clinical application.

## Author Contributions

M.H.A.S.: conceptualisation, methodology, project administration, investigation, writing – original draft. H.A.: investigation, writing – review and editing (equal). A.E.A.: investigation, writing – review and editing (equal). A.W.I.: Investigation. F.F.: writing – review and editing (equal). G.T.: methodology, visualisation, writing – review and editing (supporting). All authors gave their final approval and agreed to be accountable for all aspects of the scientific work.

## Funding

The authors have nothing to report.

## Conflicts of Interest

The authors declare no conflicts of interest.

## Supporting information


**Figure S1:** Study flow diagram depicting cohort selection and exclusions: 829 records assessed, 406 confirmed eligible and 391 included in the final analysis after excluding cases with additional augmentation after alveolar ridge preservation (*n* = 210), immediate implant placement (*n* = 64), missing health questionnaire/other key information (*n* = 97) and additional augmentation sites within the same patient (*n* = 15).
**Table S1:** Frequency of key postoperative events according to defect type. Data are presented as percentages. *p*‐values were calculated using *χ* ^2^ tests across categories.
**Table S2:** Frequency of key postoperative events according to anatomic location. Data are presented as percentages. For membrane/device exposure, incision‐line dehiscence, prolonged swelling, infection/drainage and need for additional bone grafting, global *p*‐values were obtained from likelihood‐ratio tests with ARA location modelled as a nominal four‐category variable. *p*‐values for the remaining outcomes were calculated using *χ* ^2^ tests across categories.
**Table S4:** Sensitivity‐adjusted models for postoperative complications. Values are adjusted odds ratios (ORs) with 95% confidence intervals. Sensitivity‐adjusted models included the variables retained in the main multivariable models plus age, sex, never‐smoked status, diabetes and systemic steroid us. Defect type, surgical design and flap extension were entered according to their original numerical coding scheme.
**Table S5:** Sensitivity‐adjusted Firth penalized logistic regression model for need for additional graft. Adjusted ORs were obtained by exponentiating the Firth logistic regression coefficients. The sensitivity‐adjusted model included the variables retained in the main model, namely recession on adjacent teeth, never‐smoked status and systemic steroid use, plus age, sex and diabetes.

**Appendix S1:** Supporting Information.

## Data Availability

The data that support the findings of this study are available from the corresponding author upon reasonable request.
