# Supplementary material for: Postoperative Sequelae and Complications of Alveolar Ridge Augmentation With Simultaneous Implant Placement. A Retrospective Cohort‐Based Predictive Risk Modelling
Source: J Clin Periodontol. 2026 Sep 13;53(12):1832–42. doi: 10.1111/jcpe.70196 (PMC13751317; doi:10.1111/jcpe.70196)
Supplement: Supplementary file 1 [file JCPE-53-1832-s001.docx]

**Supplemental Tables and Figures:**

**Supplemental Figure S1.** Study flow diagram depicting cohort selection and exclusions: 829 records assessed, 406 confirmed eligible, and 391 included in the final analysis after excluding cases with additional augmentation after alveolar ridge preservation (n=210), immediate implant placement (n=64), missing health questionnaire/other key information (n=97), and additional augmentation sites within the same patient (n=15).

| **Outcome** | **Horizontal (n=296)** | **Vertical (n=75)** | **Combined (n=20)** | **p-value** |
| --- | --- | --- | --- | --- |
| Membrane/device exposure | 6.1% | 21.3% | 15.0% | **0.0002** |
| Incision line dehiscence | 16.9% | 33.3% | 25.0% | **0.006** |
| Infection/drainage | 4.1% | 9.3% | 5.0% | 0.182 |
| Prolonged swelling (>2 weeks) | 18.0% | 20.0% | 25.0% | 0.701 |
| Temporary paresthesia | 1.4% | 2.7% | 5.0% | 0.404 |
| Graft loss | 3.4% | 8.0% | 5.0% | 0.216 |
| Early implant failure (pre-loading) | 2.4% | 2.7% | 0.0% | 0.771 |
| Recession on adjacent teeth | 2.4% | 5.3% | 5.0% | 0.364 |
| Screw exposure | 5.4% | 17.3% | 10.0% | **0.003** |
| Need for additional grafting | 6.8% | 4.0% | 5.0% | 0.654 |

**Supplementary Table S1.** Frequency of key postoperative events according to defect type. Data are presented as percentages. P-values were calculated using chi-square tests across categories.

| **Outcome** | **Maxillary Anterior (n=91)** | **Maxillary Posterior (n=95)** | **Mandibular Anterior (n=28)** | **Mandibular Posterior (n=177)** | **p-value** |
| --- | --- | --- | --- | --- | --- |
| Membrane/device exposure | 7.6% | 4.3% | 4.0% | 14.2% | **0.021** |
| Incision line dehiscence | 18.5% | 13.8% | 20.0% | 25.0% | 0.171 |
| Infection/drainage | 6.5% | 4.3% | 4.0% | 5.1% | 0.866 |
| Prolonged swelling (>2 weeks) | 21.7% | 19.1% | 4.0% | 19.3% | 0.097 |
| Temporary paresthesia | 1.1% | 1.1% | 0.0% | 2.8% | 0.556 |
| Graft loss | 7.6% | 2.1% | 8.0% | 3.4% | 0.204 |
| Early implant failure (pre-loading) | 4.3% | 3.2% | 0.0% | 1.1% | 0.301 |
| Recession on adjacent teeth | 5.4% | 2.1% | 0.0% | 2.8% | 0.423 |
| Screw exposure | 3.3% | 5.3% | 0.0% | 13.1% | **0.007** |
| Need for additional grafting | 10.9% | 3.2% | 16.0% | 4.0% | **0.054** |

**Supplementary Table S2.** Frequency of key postoperative events according to anatomic location. Data are presented as percentages. For membrane/device exposure, incision-line dehiscence, prolonged swelling, infection/drainage, and need for additional bone grafting, global p-values were obtained from likelihood-ratio tests with ARA location modeled as a nominal four-category variable. P-values for the remaining outcomes were calculated using chi-square tests across categories

| Model | AUC (95% CI) | Hosmer–Lemeshow test (p) | Optimal threshold | Sensitivity (%) | Specificity (%) | Accuracy (%) | LR+ | LR− |
| --- | --- | --- | --- | --- | --- | --- | --- | --- |
| Membrane Exposure | 0.708 (0.627–0.788) | 4.20 (*p* = 0.3798) | 0.0590 | 83.78 | 45.12 | 58.73 | 1.53 | 0.36 |
| Need for Additional Graft | 0.744 (0.655–0.833) | 2.31 (*p* = 0.5103) | 0.1280 | 50.00 | 82.57 | 80.30 | 2.87 | 0.61 |

**Supplemental Table S3.** Diagnostic performance of predictive models.

| **Outcome** | **Predictor** | **Adjusted OR** | **95% CI** | **p-value** |
| --- | --- | --- | --- | --- |
| Membrane exposure | Vertical/combined defects | 2.63 | 1.52–4.56 | 0.001 |
| Incision line dehiscence | Microsurgical techniques | 0.70 | 0.52–0.94 | 0.017 |
|  | Vertical/combined defects | 1.78 | 1.15–2.75 | 0.009 |
| Prolonged swelling | Flap extension (1–14 teeth) | 1.22 | 1.04–1.42 | 0.015 |
|  | Sedation (n=61) | 2.46 | 1.07–5.67 | 0.034 |

**Supplementary Table S4**. Sensitivity-Adjusted Models for Postoperative Complications. Values are adjusted odds ratios (ORs) with 95% confidence intervals. Sensitivity-adjusted models included the variables retained in the main multivariable models plus age, sex, never-smoked status, diabetes, and systemic steroid us. Defect type, surgical design, and flap extension were entered according to their original numerical coding scheme.

| **Predictor** | **Adjusted OR** | **95% CI** | **p-value** |
| --- | --- | --- | --- |
| Recession on adjacent teeth (Yes vs No) | 14.12 | 3.79–52.60 | <0.001 |
| Never smoked (Yes vs History) | 3.44 | 0.99–11.94 | 0.052 |
| Steroid use (Yes vs No) | 2.66 | 1.09–6.52 | 0.032 |

**Supplementary Table S5**. Sensitivity-Adjusted Firth Penalized Logistic Regression Model for Need for Additional Graft. Adjusted ORs were obtained by exponentiating the Firth logistic regression coefficients. The sensitivity-adjusted model included the variables retained in the main model, namely recession on adjacent teeth, never-smoked status, and systemic steroid use, plus age, sex, and diabetes.
