# Supplementary material for: Postoperative Sequelae and Complications of Alveolar Ridge Augmentation With Simultaneous Implant Placement. A Retrospective Cohort‐Based Predictive Risk Modelling
Source: J Clin Periodontol. 2026 Sep 13;53(12):1832–42. doi: 10.1111/jcpe.70196 (PMC13751317; doi:10.1111/jcpe.70196)
Supplement: Supplementary file 2 [file JCPE-53-1832-s002.docx]

A. **Appendix**

**A1. | Materials and methods**

**A.1.1. | Ethical considerations and consent**

This research complied with the Declaration of Helsinki ethical principles for human research, and the reporting followed STROBE recommendations (Cuschieri, 2019; ‘World Medical Association Declaration of Helsinki: ethical principles for medical research involving human subjects’, 2013). To protect confidentiality, all records were anonymised, and no identifiable information was accessed or stored at any stage.

**A1.2. | Eligibility criteria**

**Inclusion criteria**:

1. Presence of a deficient alveolar ridge with vertical, horizontal or combined ridge deficiency.
2. Patient having undergone ARA in conjunction with simultaneous implant placement.
3. Availability of comprehensive electronic records with detailed pre‑ and post-operative documentation.
4. Documented follow-up visits from the day of implant placement to recovery/second-stage implant surgery.

**Exclusion Criteria**

1. ARA performed at the time of extraction (immediate implant placement).
2. SARA within sites previously receiving staged ARA.
3. ARA indicated for trauma, cystic lesions or oncological resections.
4. Incomplete clinical documentation (missing graft technique, membrane information or postoperative outcomes).
5. Those lost to follow-up before the second-stage surgery.
6. Any additional sites/patient (only the first fully documented surgery/patient was included).

**A1.3. | Data Collection and Variables**

Patient records were reviewed and extracted using a standardised data abstraction form. Variables were obtained from the electronic dental records and grouped as follows:

1. **Patient-related variables.** Age at the time of procedure (years), sex and treated site identifier.

2. **Medical and behavioural history.** Tobacco use was recorded as current/former/never; for current smokers, systemic conditions were abstracted as present/absent and included diabetes mellitus, hypertension, arthritis, asthma, dialysis, stroke history, history of periodontitis, sinus problems/pathology, osteoporosis, any cancer history, lupus and mental health problems. Allergies were recorded by category (yes/no for each: penicillin, erythromycin, codeine, latex, seasonal allergies, local anaesthetics and food allergies).

3. **Medications.**  Medications routinely taken by the patient at the time of surgery were captured as present/absent, including blood thinners/anticoagulant use, history of chemotherapy and/or radiation therapy, systemic steroid use, statins, antihypertensives, antidepressants, antidiabetic and antiresorptive medications.

4. **Site and defect characteristics.** Anatomical location was coded by arch and region (maxillary anterior, maxillary posterior, mandibular anterior, mandibular posterior). Defect configuration was recorded as horizontal, vertical or combined. Defect width was categorised as ≤4 mm versus >4 mm (Pistilli et al., 2022). Defect categorisation was based on pre-op CBCT (if available, up to 6 months pre-op) or patient notes. Gingival phenotype was recorded as thin versus thick. When documented, clinical attachment loss (CAL) on teeth directly adjacent to the defect was abstracted and dichotomised as <5 mm versus ≥5 mm interdental CAL at least one adjacent site (mesial and/or distal to the grafted area).

5. **Procedure- and operator-related variables.** Operator experience was captured where applicable, and resident level/experience was recorded based on available training-year ranking. Procedure variables included the number of implants placed (count) and sedation (yes/no). Augmentation technique was recorded, including GBR, protected healing protocols using titanium mesh or titanium-reinforced PTFE membranes and block graft, as well as graft form (particulate vs. block) and whether autogenous graft material was added (yes/no). Membrane use and type were captured in detail, including specific membrane categories (e.g., collagen, titanium-reinforced, collagen + titanium-reinforced, pericardium, ACM, cross-linked collagen or no membrane). Fixation was recorded (no vs. sutures vs. pins/screws), with the fixation type specified when relevant; screw/tack fixation variables were captured as applicable (noting that certain fixation variables apply only when fixation screws and/or in simultaneous augmentation contexts). Flap/surgical design was documented, including whether vertical releasing incisions were used and the design category (gingival with one vertical, two verticals no verticals). The use of operating microscope and implementing of microsurgical techniques (Burkhardt & Lang, 2005) (with papilla-sparing) was documented (yes/no). Flap extension quantified by the number of teeth included. Adjunctive measures, including systemic antibiotics (yes/no) with antibiotic type and duration (days when documented), suture technique (simple interrupted, horizontal mattress, vertical mattress, continuous, etc.) and postoperative rinse type (none vs. chlorhexidine), were recorded.

**A1.4. AEs.** Postoperative events were recorded as present/absent and included membrane/device exposure (including exposure of mesh/plate/block graft or fixation hardware), incision-line dehiscence, prolonged swelling (≥2 weeks), infection/drainage/purulent exudate, temporary paresthesia, graft loss, erythema (≥2 weeks), tenderness (≥2 weeks), ulceration, fixation screw/tack exposure, implant exposure, early implant failure (before loading) and CAL on teeth neighbouring the defects.

AEs were defined as follows:

1. Persistent erythema: Visible redness at the surgical site that remains present at the 2-week follow-up visit.
2. Persistent tenderness: Pain or tenderness on palpation of the surgical site that continues to be present at the 2-week follow-up visit.
3. Prolonged swelling: Clinically evident facial or intraoral swelling that persists at the 2-week follow-up visit.
4. Ulceration: Superficial tissue ulceration at the grafted site that is present at the 2-week follow-up visit.
5. Fixation screw exposure: Clinical exposure of a fixation screw through the overlying soft tissue.
6. Incision-line dehiscence: Loss of primary wound closure along the incision line, resulting in separation of the wound margins, with or without membrane exposure.
7. Membrane exposure: Unintended exposure of a barrier membrane due to loss of overlying soft tissue coverage.
8. Infection: Presence of purulence and/or suppuration at the surgical site requiring clinical intervention, including antibiotic therapy and/or local treatment (flap opening and debridement).
9. Early implant failure: Loss of implant stability or removal of the implant before functional loading.
10. Graft loss or sequestration: Partial or complete loss, exfoliation or separation of the graft material from the recipient site.
11. Soft-tissue defect on adjacent teeth: New postoperative interproximal soft-tissue recession affecting a tooth adjacent to the surgical site as a result of ARA (Cairo et al., 2020).
12. Nerve injury: New postoperative sensory disturbance, including paresthesia, hypoesthesia, dysesthesia or altered sensation not attributable to concurrent swelling.

**A1.5. The need for additional grafting.** The need for additional hard- or soft-tissue grafting at any time before loading was documented.

**A2. Secondary Outcomes**

**A2.1. | Need for additional bone grafting (Secondary outcome)**

The secondary outcome included any case requiring a second regenerative procedure (bone graft) at the same site due to compromised healing or insufficient bone gain.

**A2.2. | Need for additional tissue grafting (Secondary outcome).**

Data on any cases requiring additional soft-tissue grafting at the same site were collected. Any additional soft-tissue graft procedure performed prior to crown placement was included.

**A2.3. | Prediction models** **(Secondary outcome).**

All collected data were used to develop predictive models that evaluated the contributions of local and systemic factors to (a) complication risk, (b) the need for additional bone grafting and (c) the need for additional soft-tissue grafting.

**A2.4.** | **Handling of missing data.**

Cases with missing essential information for eligibility or for primary outcome ascertainment were excluded during screening (complete-case analysis). For non-essential covariates that were incompletely documented, analyses were performed using available case data for the relevant model or variable.

**A3 | Results**

**A3.1. | Incidence of complications**

**A3.1.1. Complications by Systemic Conditions**

The overall complication rates were generally high across most systemic-condition groups (≈35%–45%) but were higher in some smaller subgroups, such as stroke (66.7%), allergy to erythromycin (55.6%), codeine allergy (55.0%), chemotherapy/radiation (54.5%), blood thinner use (52.4%) and lupus (50.0%). Membrane/device exposure and incision-line opening were frequent across many categories, while swelling was particularly notable in stroke (44.4%), food allergy (36.4%), lupus (33.3%), asthma (30.0%) and osteoporosis (26.3%). Infection/drainage was uncommon overall but appeared relatively high in osteoporosis (15.8%), blood thinner use (14.3%), stroke (11.1%), asthma (10.0%) and mental health problems (10.0%). Need for additional bone grafting was low in most groups, but numerically higher in erythromycin allergy (22.2%), stroke (11.1%), latex allergy (11.1%), codeine allergy (10.0%) and blood thinner use (9.5%), whereas the need for soft-tissue grafting showed broader variability and was highest in chemotherapy/radiation (36.4%), erythromycin allergy (33.3%), blood thinner use (28.6%), asthma (27.5%), food allergy (27.3%), osteoporosis (26.3%) and lupus (25.0%).

**A3.1.2. Complications by Medication Categories**

Across the medication categories, overall complication rates were generally moderate to high, with the highest values seen in patients taking blood thinners (54.8%) and chemotherapeutics (54.5%), followed by antidepressants (44.8%), whereas lower rates were seen with anti-diabetics (25.0%) and anticonvulsants (28.6%). Incision-line opening was common across several groups, especially blood thinners (29.0%), statins (25.5%) and antihypertensives (25.0%). Swelling was particularly prominent among antidepressant users (37.9%), and this same group also showed the highest rates of infection/drainage (17.2%), graft loss (13.8%), early implant failure (6.9%), recession on adjacent teeth (10.3%) and fixation screw exposure (20.7%). The need for additional bone grafting was low across all medication groups and absent in several, while the need for soft-tissue grafting was highest among patients on chemotherapeutics (36.4%), antidepressants (34.5%) and blood thinners (29.0%).

**A3.1.3. Complications by Anatomic and Procedural Variables**

Across the operator- and procedure-related variables, complication rates appeared to vary mainly by defect configuration, anatomic location, membrane/device type, flap design and selected procedural adjuncts. Vertical and combined defects showed less favourable descriptive profiles than horizontal defects, with higher membrane/device exposure (21.3% and 15.0% vs. 6.1%), incision-line opening (33.3% and 25.0% vs. 16.9%) and total complications (54.7% and 60.0% vs. 35.8%), while vertical defects also showed higher soft-tissue grafting needs (25.3%). Descriptively, total complication rates were relatively high at maxillary anterior and mandibular posterior sites (43.5% and 42.4%, respectively), while mandibular anterior sites had the highest proportion requiring additional bone grafting (16.0%). These descriptive findings should not be interpreted as adjusted associations. When ARA location was modelled as a nominal four-category variable, it was significantly associated overall only with membrane/device exposure (global likelihood-ratio *p* = 0.0218), but not with incision-line dehiscence (*p* = 0.1712), prolonged swelling (*p* = 0.0973), infection/drainage (*p* = 0.8666) or additional bone grafting (*p* = 0.0548). Sedation was associated with higher swelling (31.1%), total complications (49.2%) and soft-tissue grafting (27.9%). Use of two vertical releasing incisions was associated with higher total complications (44.9%) and additional grafting (14.5%) than no vertical release or one vertical release. The most striking descriptive findings involved device/material categories: Ti-reinforced PTFE showed very high rates of membrane exposure, incision-line opening and both additional bone and soft-tissue grafting (all 66.7%), although this likely reflects a very small subgroup and should be interpreted cautiously; pericardium and autogenous scraping also showed relatively high overall complication frequencies (50.8% and 51.3%). Fixation-related differences were less consistent overall, although pin/screw fixation showed a high need for additional bone grafting (18.2%).

**A3.2. | Infection**

Postoperative infection emerged as a relatively infrequent event. No patient-, defect- or technique-related variable showed any significant association with infection. Univariable analysis demonstrated that infection doubled the odds of each additional postoperative day of antibiotics (OR 2.22; 95% CI: 1.38–3.58; *p* = 0.001). (Table 2)

**A3.3. | Prolonged swelling.**

Across the cohort, the persistence of postoperative oedema was primarily influenced by intraoperative management and the size of the surgical field. Univariable analysis identified several signals: cases performed under sedation showed a more than two-fold increase in risk (OR 2.37; CI: 1.28–4.41; *p* = 0.006). Both heavy smokers and non-smokers increased the odds of swelling (OR 1.52; CI: 1.00–2.31; *p* = 0.048 and OR 1.87; CI: 1.05–3.34; *p* = 0.035, respectively). Each additional tooth site incorporated into the flap expanded the risk by about one-fifth (OR 1.21 per tooth site; CI: 1.04–1.40; *p* = 0.010), whereas male sex appeared protective, reducing the likelihood by more than half (OR 0.44; CI: 0.25–0.76; *p* = 0.003).

When all covariates were entered into the multivariable model, only flap extension and the use of sedation retained independent weight. Each additional tooth site reflected in the flap continued to add a 21% rise in risk (adjusted OR 1.21; CI: 1.04–1.39; *p* = 0.012), and sedation remained associated with more than twice the probability of prolonged swelling (adjusted OR 2.31; CI: 1.09–4.87; *p* = 0.028) (Figure 3) (Table 2).

**A3.4 | Need for additional soft-tissue grafting.**

Only 373 sites had this available outcome data; 76 (20.4%) required additional soft-tissue grafting before crown placement. In univariable logistic regression, antidepressant use was associated with the outcome (OR 2.35; 95% CI, 1.04–5.32; *p* = 0.041), while sedation (OR 1.91; 95% CI, 1.01–3.60; *p* = 0.047) and systemic steroid use (OR 1.64; 95% CI: 0.96–2.79; *p* = 0.070) showed weaker or borderline associations. When these variables were entered simultaneously into the multivariable model, antidepressant use (OR 2.13; 95% CI: 0.91–4.97; *p* = 0.080), sedation (OR 1.81; 95% CI: 0.92–3.56; *p* = 0.087) and systemic steroid use (OR 1.34; 95% CI: 0.76–2.37; *p*= 0.309) did not retain statistical significance.

**A4 | Discussion**

**A4.1. Main findings**

Early membrane exposures are typically a result of incision-line dehiscence, whereas late exposures are typically associated with wound breakdown within the augmentation area (facial, oral or crestal) and are much more frequently observed with rigid devices (Alotaibi, Rocchietta, Buti, & D'Aiuto, 2023; Elrefaei et al., 2025; Jung, Halg, Thoma, & Hammerle, 2009). Such exposures are often associated with insufficient soft-tissue coverage, excessive flap tension or compromised vascularisation (Urban et al., 2024), which may impair regenerative outcomes and increase the risk of bacterial contamination (Hindryckx et al., 2025; Sbricoli et al., 2020). In the present cohort, exposure rates reached up to 50% in vertical or combined defects, consistent with previous reports (Chiapasco & Zaniboni, 2009; Sanz et al., 2019). A previous study reported that early membrane removal was necessary in 15.4% of cases, with 78.6% of cases occurring in SARA (Zeller et al., 2024).

**A4.2. Limitations**

In this study, second-stage surgeries were not standardised specifically to evaluate the need for additional ARA. In routine clinical practice, the second stage is often performed through a limited-access incision to place a healing abutment without elevating an extensive flap. However, in this training environment, flap reflection is common, allowing residents to directly assess the outcomes of the augmentation procedure and document healing.

Nonetheless, within these limitations, the findings emphasise the importance of individualised, anatomy-driven risk assessment in ARA and that refined predictive models may support the implementation of precision medicine in implant dentistry (Buser, Urban, Monje, Kunrath, & Dahlin, 2023). From a clinical standpoint, the presented models may provide a practical framework for individualised preoperative risk stratification but may benefit from integrating machine-learning approaches (Troiano et al., 2023). If externally validated, these models could serve as point-of-care tools that enhance consistency in treatment planning and support a more personalised approach to SARA. Finally, the WOUND framework introduced here may benefit from further stratification and validation before being used in practice.

References

Alotaibi, F. F., Rocchietta, I., Buti, J., & D'Aiuto, F. (2023b). Comparative Evidence of Different Surgical Techniques for the Management of Vertical Alveolar Ridge Defects in Terms of Complications and Efficacy: A Systematic Review and Network Meta-Analysis. *Journal of Clinical Periodontology, 50*(11), 1487–1519. doi:[10.1111/jcpe.13850](https://doi.org/10.1111/jcpe.13850).

Burkhardt, R., & Lang, N. P. (2005b). Coverage of Localized Gingival Recessions: Comparison of Micro- and Macrosurgical Techniques. *Journal of Clinical Periodontology, 32*(3), 287–293. doi:[10.1111/j.1600-051X.2005.00660.x](https://doi.org/10.1111/j.1600-051X.2005.00660.x).

Buser, D., Urban, I., Monje, A., Kunrath, M. F., & Dahlin, C. (2023b). Guided Bone Regeneration in Implant Dentistry: Basic Principle, Progress Over 35 Years, and Recent Research Activities. *Periodontology 2000, 93*(1), 9–25. doi:[10.1111/prd.12539](https://doi.org/10.1111/prd.12539).

Cairo, F., Nieri, M., Cavalcanti, R., Landi, L., Rupe, A., Sforza, N. M.,. .. Barbato, L. (2020b). Marginal Soft Tissue Recession After Lateral Guided Bone Regeneration at Implant Site: A Long-Term Study With at Least 5 Years of Loading. *Clinical Oral Implants Research, 31*(11), 1116–1124. doi:[10.1111/clr.13658](https://doi.org/10.1111/clr.13658).

Chiapasco, M., & Zaniboni, M. (2009b). Clinical Outcomes of GBR Procedures to Correct Peri-Implant Dehiscences and Fenestrations: A Systematic Review. *Clinical Oral Implants Research, 20 Suppl 4*, 113–123. doi:[10.1111/j.1600-0501.2009.01781.x](https://doi.org/10.1111/j.1600-0501.2009.01781.x).

Cuschieri, S. (2019b). The STROBE Guidelines. *Saudi J Anaesth, 13*(Suppl 1), S31–s34. doi:[10.4103/sja.SJA_543_18](https://doi.org/10.4103/sja.SJA_543_18).

Hindryckx, M., De Bruyckere, T., De Buyser, S., Seyssens, L., Shtino, R., Younes, F., & Cosyn, J. (2025b). A Multi-Centre Randomised Controlled Trial Comparing dPTFE Membrane to Collagen Membrane in Lateral Bone Augmentation at Single Sites in the Anterior Maxilla: 1-Year Results. *Journal of Clinical Periodontology, 52*(8), 1136–1146. doi:[10.1111/jcpe.14174](https://doi.org/10.1111/jcpe.14174).

Jung, R. E., Halg, G. A., Thoma, D. S., & Hammerle, C. H. (2009b). A Randomized, Controlled Clinical Trial to Evaluate a New Membrane for Guided Bone Regeneration Around Dental Implants. *Clinical Oral Implants Research, 20*(2), 162–168. doi:[10.1111/j.1600-0501.2008.01634.x](https://doi.org/10.1111/j.1600-0501.2008.01634.x).

Pistilli, R., Barausse, C., Simion, M., Bonifazi, L., Karaban, M., Ferri, A., & Felice, P. (2022b). Simultaneous GBR and Implant Placement With Resorbable Membranes in the Rehabilitation of Partially Edentulous and Horizontally Atrophic Dental Arches: A Retrospective Study on 97 Implants With a 3- To 7-Year Follow-Up. *The International Journal of Periodontics & Restorative Dentistry, 42*(3), 371–379. doi:[10.11607/prd.5641](https://doi.org/10.11607/prd.5641).

Sanz, M., Dahlin, C., Apatzidou, D., Artzi, Z., Bozic, D., Calciolari, E.,. .. Schliephake, H. (2019b). Biomaterials and Regenerative Technologies Used in Bone Regeneration in the Craniomaxillofacial Region: Consensus Report of Group 2 of the 15th European Workshop on Periodontology on Bone Regeneration. *Journal of Clinical Periodontology, 46 Suppl 21*, 82–91. doi:[10.1111/jcpe.13123](https://doi.org/10.1111/jcpe.13123).

Sbricoli, L., Guazzo, R., Annunziata, M., Gobbato, L., Bressan, E., & Nastri, L. (2020b). Selection of Collagen Membranes for Bone Regeneration: A Literature Review. *Materials (Basel), 13*(3). doi:[10.3390/ma13030786](https://doi.org/10.3390/ma13030786).

Troiano, G., Nibali, L., Petsos, H., Eickholz, P., Saleh, M. H. A., Santamaria, P.,. .. Ravida, A. (2023b). Development and International Validation of Logistic Regression and Machine-Learning Models for the Prediction of 10-Year Molar Loss. *Journal of Clinical Periodontology, 50*(3), 348–357. doi:[10.1111/jcpe.13739](https://doi.org/10.1111/jcpe.13739).

Urban, I. A., Saleh, M. H. A., Serroni, M., Shahbazi, A., Baksa, G., Szoke, P., & Ravida, A. (2024b). Management of the Lingual Flap During Vertical Augmentation of the Atrophic Anterior Mandible: Anatomical Overview and Description of the Technique. *The International Journal of Periodontics & Restorative Dentistry, 44*(1), 17–25. doi:[10.11607/prd.6667](https://doi.org/10.11607/prd.6667).

World Medical Association Declaration of Helsinki: Ethical Principles for Medical Research Involving Human Subjects. (2013b). *Jama, 310*(20), 2191–2194. doi:[10.1001/jama.2013.281053](https://doi.org/10.1001/jama.2013.281053).

Zeller, A. N., Schenk, R., Bonsmann, M., Stockbrink, G., Becher, S., & Pabst, A. (2024b). Complication Rates of Guided Bone Regeneration Using Titanium-Reinforced PTFE Membranes: A Retrospective Analysis. *Clinical Oral Investigations, 28*(11), 616. doi:[10.1007/s00784-024-06007-4](https://doi.org/10.1007/s00784-024-06007-4).
